# Nursing informatics skills relevance and competence for final year nursing students

**DOI:** 10.4102/curationis.v45i1.2277

**Published:** 2022-11-21

**Authors:** Jennifer Chipps, Loretta le Roux, Jakobina Agabus, Million Bimerew

**Affiliations:** 1 Faculty of Community and Health Sciences, School of Nursing, University of the Western Cape, Cape Town, South Africa

**Keywords:** attitudes, competence, nursing informatics skills, final year nursing student, information technology, nursing practice

## Abstract

**Background:**

The increasing use of technology in nursing practice requires nursing students to be competent in nursing informatics with an attitude of acceptance of technology in the healthcare environment.

**Objectives:**

The objectives of the study were to determine final year nursing students’ perceptions and skills in nursing informatics and their attitudes towards computerisation in nursing practice.

**Method:**

The study population were 198 final year nursing students from a selected university in the Western Cape, South Africa. All-inclusive sampling was used. A descriptive survey was conducted using a self-administered questionnaire which included two validated scales, namely the validated Nursing Informatics Competency Assessment Tool (NICAT) and the Nurses’ Attitudes towards Computerisation scale. Means and 95% confidence intervals (CI) of the ratings of the perceived relevance of nursing informatics skills in nursing practice, perceived levels of competence in nursing informatics skills and attitudes towards computers were calculated.

**Results:**

A total of 91 undergraduate respondents completed the survey. Computer literacy skills were rated overall as most relevant (4.23, 95% confidence interval [95% CI]: 4.06–4.40) and the skills perceived most competent (4.16, 95% CI: 3.81–4.22). The respondents had an overall positive score for attitudes towards computerisation in healthcare (67.34, s.d. = 10.40, 95% CI: 65.18–69.51).

**Conclusion:**

The study concluded that computer literacy skills, informatics literacy skills and information management skills were relevant to nursing practice, despite varying levels of competence in these skills among nurses.

**Contribution:**

What key insights into the research results and its future function are revealed? How do these insights link to the focus and scope of the journal? It should be a concise statement of the primary contribution of the manuscript; and how it fits within the scope of the journal.

## Introduction

The Fourth Industrial Revolution (4IR) has a major influence on the health sector, with innovative digital changes to treatment, diagnosis and monitoring of patients (Araújo 2020). Especially in this context, nursing informatics has been identified as an enabler to facilitate the delivery of nursing care, education and administration (Akpabio & Ella 2015; Green et al. 2016; Hussey & Kennedy 2016; Shin, Cummings & Ford 2018). Nursing Informatics Science is the management and processing of health and nursing data, and information through the application of computers and Information Communication Technology (ICT) (Hübner et al. 2018). The use of nursing informatics can contribute to patient safety, quality of healthcare, can reduce healthcare costs (Jouparinejad et al. 2020), fosters patient trust and leads to improved nursing outcomes.

Even though the relevance of nursing informatics in nursing practice has been widely documented through global research, the integration of nursing informatics in nursing education and the perceived relevance to nursing practice for nursing students have not been widely studied. Studies have shown that although newly graduated nurses are aware of the need to use information systems and skills in practice, they were not sufficiently computer-literate and had insufficient understanding of healthcare information technology to meet nursing practice requirements (Gürdaş Topkaya & Kaya 2015; Vasuki 2016). In addition, although nursing students learn to engage with technology during their educational programme, studies reported variable skills when it comes to technology use, and evidence that they may not necessarily regard it as relevant to clinical practice (Harerimana et al. 2020; Levett-Jones et al. 2009).

As nursing students are in the practice environment during clinical placements, there is an expectancy that they should be proficient in ICT (Lee & Clarke 2015). The digital era requires nurses who are competent to use data, information and technologies effectively for the improvement of nursing care (Hübner et al. 2018). However, nursing informatics incompetence of nurses can result in less than optimal patient care (Jouparinejad et al. 2020), which can result in sub-optimal healthcare outcomes, such as decreased patient safety and errors in clinical practice (Konttila et al. 2019; Rajalahti, Heinonen & Saranto 2014). Similarly, nurses’ attitude towards the use and acceptance of technology in nursing practice may affect their competency in nursing informatics and their adoption of healthcare technology (Heidarizadeh et al. 2017; Kaminski 2010; Mutula 2015). Studies have shown that older nurses, their educational level, the number of years worked in nursing, their computer experience (Kipturgo et al. 2014), and fear of technology, may lead to negative attitudes towards technology use (Fagerström et al. 2017; Gürdaş Topkaya & Kaya 2015; Heidarizadeh et al. 2017). However, these attitudes could be positively influenced with increased experience in computer use and understanding the importance of information technology in healthcare (Fagerström et al. 2017; Heidarizadeh et al. 2017).

To improve nursing informatics competence, it is important that nursing curricula link the use of information technologies for educational purposes and the use in clinical practice (Forman, Armor & Miller 2020). In the study setting, the formal inclusion of nursing informatics in nursing education was deemed to be inadequate, with a concomitant limited usage of informatics tools in the clinical settings (Willemse, Jooste & Bozalek 2019). It was hypothesised that a lack of formal nursing informatics training may result in students with negative attitudes towards informatics because of low informatics competency and a poor understanding of its relevance for nursing practice (Harerimana et al. 2020; Willemse et al. 2019). In addition, the lack of various health information technologies in practice, limits their exposure to informatics in practice (South African National Department of Health 2019). This study aimed to investigate undergraduate nursing students’ perceptions of the relevance and their competence of nursing informatics and their attitudes towards informatics use in nursing practice.

## Methods

A descriptive survey, using a self-administered questionnaire, was conducted to investigate the perceived relevance, competence and attitudes towards nursing informatics skills of final year undergraduate nursing students. The study was conducted in a selected school of nursing at a university in the province of the Western Cape in South Africa. The school offers a 4-year undergraduate nursing degree and various postgraduate programmes. Although the nursing students are exposed to a variety of educational technologies such as online learning and high-fidelity simulation during their training, no formal informatics training for nursing practice is offered in the programme.

### Study population and study sample

The targeted population for this study were the 198 final year undergraduate nursing students enrolled in a 4-year undergraduate nursing programme. All-inclusive sampling was used with questionnaires distributed to all 198 students. Final year students were chosen as these students were at the end of their student training and were entering formal nursing practice on graduation.

### Instrument

A self-administered questionnaire based on the validated Nursing Informatics Competency Assessment Tool (NICAT) (Rahman 2015) and the Nurses’ Attitudes towards Computerisation (NATC) (Stronge & Brodt 1985) was used with permission from the authors. The NICAT has 30 items reflecting nursing informatics skills in three domains (computer literacy, informatics literacy and informatics management) (Rahman 2015). Each item is rated for perceived relevance (relevant [1] to extremely relevant [5]) and perceived competence (competent [1] to expert [5]) (Rahman 2015). The NATC scale included 20 attitudinal statements (six positive and 14 negative statements) rated from strongly disagree (1) to strongly agree (2) (Stronge & Brodt 1985). The questionnaire was in English language, as English is the academic medium in the university, and except for minor contextual word changes, no changes were made to the scales. The questionnaire was pretested on five nursing students to identify the consistency, acceptability and ambiguity in the questions. No changes were made to the questions other than the minor language corrections made on some questions. The NICAT subscales of relevance (α = 0.973) and competence (α = 0.937) had good internal consistency, with moderate consistency for the NATC positive statements (α = 0.718) and negative statements (α = 0.851).

### Data collection

Data collection was carried out by a trained research assistant during the months of October 2017 and November 2017. To ensure the availability of all students and enough time to complete the questionnaire, the research assistant met the fourth-year nursing students after a class session on a date and time provided by the class lecturers. The self-administered anonymous questionnaires, along with the information sheet and consent, were handed out to all students. An explanation of the study aim was provided, and it was reiterated that the completion of the questionnaires was voluntary and anonymous. The questionnaire took 20 min to complete and was submitted in a box as they exited the classroom.

### Data analysis

Data analysis was conducted with SPSS^®^ version 27. Average scores with 95% confidence intervals (CI) for perceived relevance and perceived competence were calculated for all the informatics items in the NICAT and for the three domains of computer literacy, informatics literacy and informatics management. Pearson’s correlation analysis was done to assess correlation among the domains and attitudes. An average score with 95% CI was also calculated for the negative and positive attitudinal statements and an overall attitudes score (out of 100) was calculated after negative rated items were reversed.

### Ethical considerations

Ethics approval was obtained from the University of the Western Cape Humanities and Social Sciences Research Ethics Committee with Ethics reference number: HS17/1/27, and permission to conduct the study was obtained from the university registrar, the head of the nursing school and the relevant lecturer.

## Results

A total of 91 (45.9%) of the final year nursing respondents completed the survey. Nearly three-quarters of the respondents were female (65, 71.4%). The ages of respondents ranged between 19 and 49 years with over three-quarters (72, 79.1%) aged between 21 and 30 years (average age 25.8 [±5.6] years). Out of the 91 respondents, only 26 (28.6%) respondents reported that they had attended formal computer training and only four (4.4%) reported that they had attended informatics training.

### Overall nursing informatics skills

**FIGURE 1:**
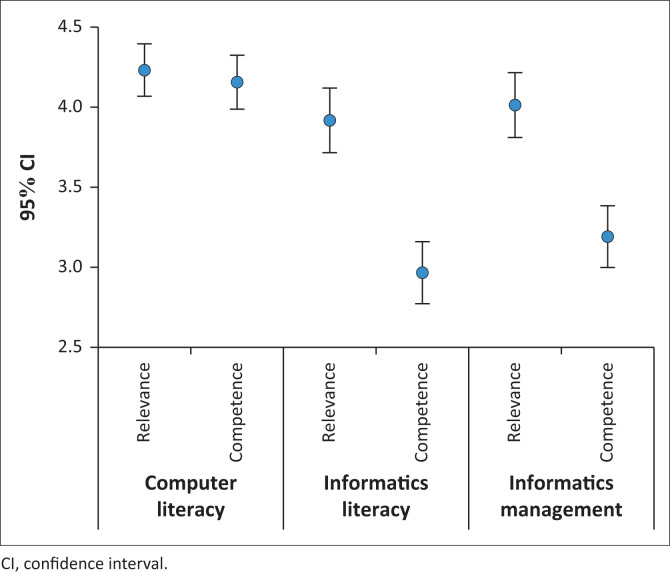
Average scores for nursing informatics skills.

Nursing informatics skills were measured in terms of perceived relevance and perceived competence in three areas: Computer literacy skills, Informatics literacy skills and Informatics management skills (Figure 1).

Computer Literacy was rated as most relevant (4.2, 95% CI: 4.1–4.4), followed by Informatics Management (4.0, 95% CI: 3.8–4.2), with Informatics Literacy rated as least relevant (3.9, 95% CI: 3.7–4.1), though the ratings were not significantly different, Computer Literacy competence (4.2, 95% CI: 4.04.3) was rated significantly higher than Informatics Management (3.2, 95% CI: 3.0–3.4) and Informatics Literacy which was rated the lowest (3.0, 95% CI: 2.8–3.2). For both Informatics Management and Informatics Literacy, competence was significantly rated lower than relevance (Figure 1). No significant differences were observed in any of these areas for either relevance or competence for gender or previous training.

Strong significant correlations were found between computer literacy and informatics literacy relevance (*r* = 0.587, *p* < 0.001), computer literacy and informatics management relevance (*r* = 0.587, *p* < 0.001) and informatics literacy and informatics management relevance (*r* = 0.873, *p* < 0.001). Only one strong significant correlation was found for competence between informatics literacy and informatics management (*r* = 0.742, *p* < 0.001). Weak correlations were found between relevance and competence among the three areas (*p* < 0.05), with weak correlation between computer literacy relevance and informatics literacy competence being not significant (NS) (0.167).

### Computer literacy skills

Computer literacy skills were rated overall as most relevant (4.23, 95% CI: 4.06–4.40) and the skills perceived most competent (4.16, 95% CI: 3.81–4.22) (Figure 1). The highest rated computer literacy skills in terms of relevance and competence were related to using Microsoft office and word processing (Table 1).

**TABLE 1:** Computer literacy skills.

Computer literacy	Relevance		Competence	
	Mean	95% CI	Mean	95% CI
Create, rename, move and delete files using computer operating systems such as Microsoft Windows	4.56	4.17–4.56	4.51	4.13–4.51
Use word processing function such as save, categorise documents, copy, paste and delete	4.54	4.17–4.54	4.52	4.14–4.52
Use of telecommunication tools such as electronic mail and facsimile (fax)	4.51	4.17–4.51	4.43	4.01–4.43
Recognise the basic components of the computer system such as mouse, screen and workstation	4.51	4.13–4.51	4.50	4.07–4.5
Use software to create presentations such as Microsoft PowerPoint	4.51	4.13–4.51	4.44	4.07–4.44
Use external devices such as USB flash drive, digital camera, CDROM	4.46	4.05–4.46	4.36	3.97–4.36
Use of remote communication tools such as Skype, Zoom, WhatsApp, Hangout, etc.	4.39	3.94–4.39	4.39	4.00–4.39
Perform basic computer systems troubleshooting such as checking power source, rebooting computer and printing	4.39	3.94–4.39	4.23	3.75–4.23
Manage computer systems security to protect data, devices and passwords	4.25	3.79–4.25	4.07	3.58–4.07
Navigate computer operating systems to access installed applications and choose active printers	4.23	3.79–4.23	4.21	3.75–4.21

*Source*: Nursing Informatics Competency Assessment Tool, Rahman, A., 2015, *Development of a nursing informatics competency assessment tool (NICAT)*, Walden University

CI, confidence intervals.

### Informatics literacy

Informatics literacy skills were rated overall as the least relevant (3.91, 95% CI: 4.06–4.40, NS) and the respondents perceived themselves to be significantly less competent in the informatics skills (2.97, 95% CI: 2.77–3.12) (Figure 1). The highest rated informatics literacy skills in terms of relevance and competence were related to using the Internet (Table 2). Using medication administration and dispensing systems were rated as least relevant and the skills in which they had the lowest perceived competence (Table 2).

**TABLE 2:** Informatics literacy skills.

Informatics literacy skills	Relevance		Competence	
	Mean	95% CI	Mean	95% CI
Use the Internet to locate and download items of interest	4.56	3.71–4.12	4.57	4.17–4.57
Navigate patients’ electronic health records	4.38	3.91–4.38	3.02	2.41–3.02
Review and acknowledge patient orders in an electronic health record	4.31	3.78–4.31	2.97	2.37–2.97
Collect and electronically document patient signs, height and weight	4.25	3.75–4.25	3.73	3.15–3.73
Continue patient care documentation and patient identification when computer system is down	4.21	3.74–4.21	3.49	2.98–3.49
Use electronic systems to assist with admission and discharge process	4.19	3.72–4.19	3.39	2.92–3.39
Develop and document care plan in electronic health record	4.21	3.70–4.21	2.81	2.21–2.81
View trended electronic documentation to understand the effectiveness of nursing interventions	4.16	3.69–4.16	3.41	2.94–3.41
Review electronic point of care data such as urine monitoring data relevant to care such as vitals dipstick, glucose check, and haemoglobin meter, bloods to make timely decisions	4.16	3.64–4.16	3.09	2.49–3.09
Respond appropriately to alerts from clinical decision-making tools such as algorithms, best practice alerts	4.01	3.48–4.01	2.91	2.35–2.91
Conduct literature searches in the accessible proprietary database systems such as CINAHL, EBSCO, etc.	4.00	3.47–4.00	3.19	2.61–3.19
Use medication administration tools such as barcode medication verification and scanning	3.90	3.33–3.9	2.86	2.30–2.86
Use of medication dispensing system or other electronic pharmacy dispensing units	3.87	3.3–3.87	2.60	2.10–2.60

*Source*: Nursing Informatics Competency Assessment Tool, Rahman, A., 2015, *Development of a nursing informatics competency assessment tool (NICAT)*, Walden University

CI, confidence intervals.

### Information management skills

Information management skills were rated overall as relevant (4.01, 95% CI: 3.8–4.21) and respondents perceived themselves to be less competent in information management skills than computer literacy skills, but more competent than informatics literacy skills (3.19, 95% CI: 3.00–3.39) (Figure 1). The highest rated information management skills in terms of relevance and competence were related to confidential data management (Table 3).

**TABLE 3:** Information management skills.

Information management	Relevance		Competence	
	Mean	95% CI	Mean	95% CI
Protect confidential patient data by logging out, suspending sessions and password protection	4.34	3.92–4.34	3.67	3.21–3.67
Use nursing data for improving practice and for clinical decision-making.	4.33	3.89–4.33	3.60	3.14–3.60
Use data and statistical reports for unit-based quality improvement initiatives and practice evaluation.	4.32	3.85–4.32	3.49	3.01–3.49
Find information stored in the clinical information system to guide patient care such as standardised care plans and guidelines	4.27	3.84–4.27	3.62	3.17–3.62
Use electronic communication with colleagues, patients, or other departments	4.20	3.74–4.20	3.54	3.06–3.54
Use electronic health record and other clinical information system as per organisational policy for documentation.	4.13	3.65–4.13	3.00	2.43–3.00
Use information technology as a primary means of patient safety such as bedside laboratory verification, barcode scanning, etc.	4.11	3.58–4.11	3.14	2.60–3.14

*Source*: Nursing Informatics Competency Assessment Tool, Rahman, A., 2015, *Development of a nursing informatics competency assessment tool (NICAT)*, Walden University

CI, confidence intervals.

### Attitudes towards computerisation

The total attitude scores for respondents were 67.34 (s.d. = 10.40, 95% CI: 65.18–69.51) out of a maximum possible score of 100. The range of attitude scores for this sample was 40–94. Generally, the nurses had a positive attitude towards computerisation with the highest scores for reducing workload and increasing efficiency (Table 4). No significant correlations were found between attitudes and any of the three areas for relevance or competence.

**TABLE 4:** Attitudes towards computerisation.

	Agreement level/5	95% CI
**Positive attitudes**		
Paperwork for nurses can be reduced greatly using computers	3.90	3.70–4.10
Computers save steps and allow the nursing staff to become more efficient	3.70	3.53–3.87
Computers make nurses’ jobs easier	3.55	3.33–3.77
Computerisation of nursing data offers nurses a remarkable opportunity to improve patient care	3.55	3.34–3.76
Increased computer use will allow nurses more time to give patient care	3.22	3.00–3.44
Nursing data cannot be manipulated using computers	2.90	2.67–3.13
**Negative attitudes**		
Computers contain too much personal data to be used in an area as open as a nursing station	3.26	3.02–3.5
Orientation for new employees takes longer because of computers	2.93	2.68–3.18
Only one person at a time can use a computer terminal and, therefore, staff efficiency is inhibited.	2.92	2.69–3.15
Computers can cause nurses to give less time to quality nursing care	2.89	2.67–3.11
Confidentiality will be sacrificed by patient records being computerised	2.88	2.64–3.12
The more computers in an institution, fewer jobs for employees	2.88	2.62–3.14
Computers can cause a decrease in communication between hospital departments	2.78	2.54–3.02
Because of computers, nurses will face more lawsuits	2.70	2.49–2.91
Costs of healthcare are likely to increase because of computers	2.70	2.47–2.93
The time spent using computers in health is out of proportion to the benefits	2.55	2.32–2.78
Use of computers in healthcare increases costs by increasing the nurses’ workload	2.50	2.25–2.75
Computers represent a violation of patient privacy	2.33	2.10–2.56
If I had my way, nurses would never have to use computers	2.17	1.94–2.40
Computers should only be used in the financial department	2.04	1.81–2.27

*Source*: NATC Stronge, J.H. & Brodt, A., 1985, ‘Assessment of nurses’ attitudes toward computerization’, *Computers in Nursing* 3(4), 154–158

CI, confidence intervals.

## Discussion

This study investigated the perceived relevance and competence of computer literacy, informatics literacy and informatics management in final year nursing students. In addition, attitudes to computerisation were also investigated.

### Computer literacy

Overall, computer literacy skills were rated the highest for relevance and competence by the respondents with the highest rated computer literacy skill being using Microsoft Office and Word processing and the lowest relevance and competence ratings for skills to navigate computer operating systems. The high ratings for computer literacy skills, and specifically the high ratings for the use of Word processing, were likely because of the use of Word processing and computers for educational tasks during their training and the use of social media and email to communicate socially and educationally. This is also confirmed in other studies where students are reported to commonly make use of smart phone technologies and computers at home, communicate daily via email, the Internet and social media platforms, complete assignments through the use of computers, and many have received computer training as part of nurse training (Elewa & El Guindy 2017). Telecommunication tools such as email, facsimiles (fax), WhatsApp groups and Zoom meetings are also used commonly in hospitals, making it possible that exposure of respondents to these skills in the clinical environment may also have enhanced their perceived relevance of these skills (Vasuki 2016). The use of telecommunication tools in the clinical setting allows clinicians to communicate regarding patient care (Rincon & Henneman 2018), allows better patient monitoring for adverse events, and facilitates compliance with best practices (Koivunen & Saranto 2018). Although computer training and Word processing may be included in undergraduate training, the findings indicate that there may be a need for more in-depth education and support in the use of contemporary computer technologies, other than Word processing, within the healthcare environment (Mills et al. 2015).

### Informatics literacy

Informatics literacy skills for use in nursing practice were rated overall as the least relevant and the respondents perceived themselves to be significantly less competent in informatics literacy skills. The modern healthcare environment requires nurses who are capable of successfully implementing clinical information systems, tools and devices (Khezri & Abdekhoda 2019), which further emphasises the importance of nurse education in keeping pace with informatics-related changes and the implications of this for students in practice (Kinnunen et al. 2017). The lowest ratings were for smart devices for medication management. These types of systems are not generally available in most clinical practice settings where the study participants were placed for clinical practice, thus explaining the low ratings of relevance and perceived competence. In the new 4IR, smart devices for medication are just one of many new innovations. The relevance and competence of learning to use new innovative devices, should be a specific focus for nursing educators as learning to use new devices should be integrated into the daily work of nurses to give them enough time, opportunities and resources to adapt to new technologies or to learn how to use new technologies competently (Konttila et al. 2019).

Although informatics literacy skills were rated the lowest, the highest rated informatics literacy skills were related to using the Internet. The respondents’ involvement in the accessing of research findings, both in the educational and the clinical environment, the documenting of searches as a curriculum requirement in their educational programme, and the exposure to a variety of virtual tools and online environments such as eLearning within their educational programme, could have yielded a greater familiarity with the use of the Internet and Internet-related software and could have been a potential reason why their perceived relevance ratings for this skill were higher. Even so, it is significant to note that previous studies have reported that even though nursing students were frequent Internet users and using the Internet for almost all their study purposes, they had poor literacy skills with regard to the finding of credible and reliable information and were unable to evaluate high-quality from low-quality health resources on the Internet (Rathnayake & Senevirathna 2019), thus highlighting the complexity of informatics literacy skills.

### Information management skills

Information management skills in nursing practice were rated overall as relevant, although respondents perceived themselves to be less competent in information management than in computer literacy, but more than informatics literacy. The highest rated information management skill was the confidential data management and using nursing data for improved practice and decision-making in nursing practice. The high ratings in these two skills may relate to the dominance of these skills in nursing curricula. The teaching of confidentiality of patient data is integral in most nursing curricula and the use of data for improved practice and decision-making may be related to the focus on research skills in nurse training to access to evidence-based information. Sound decision-making for improved nursing practice is a prerequisite for evidence-based practice, and is dependent on the skill to retrieve information (Rajalahti et al. 2014) and accessing research findings, both in the education and workplace (Mills et al. 2015). This study highlights the importance for nurses to be able to access data to ensure that nursing practice is informed by best practices based on evidence from research (Mokhtar et al. 2012)

A concern was that respondents rated using electronic health records as less relevant and reported lower ratings of perceived competence. In the current climate, the era of paper-based systems for documenting patient care is drawing to an end, and the use of the electronic health record for all documentation practice will become mandated (Bowling 2016). As early as in 2014, expertise in electronic documentation has been highlighted as an important challenge in healthcare (Rajalahti et al. 2014) and still remains so today in this study setting. This finding may also reflect the status of electronic record-keeping in South Africa, where most of the clinical facilities still use paper records and electronic record data are used for patient registration and only health facility level data routinely collected using District Information Systems (Maïga et al. 2019). A study on the use of electronic health information systems in South Africa showed that the most common role was the support of services such as radiology and pathology and evaluation and administrative purposes with few systems that support patient clinical care (Wright, O’Mahony & Cilliers 2017). In addition, within the healthcare facilities where the respondents were placed for clinical practice, uploading of patient information into the electronic health record systems is an administrative duty and patient medical records are still kept in filing cupboards (Modise, Jantjies & Mavetera 2019). Thus, even though South Africa has started to prioritise ICT to improve health services provision, the public health system lacks a functional health information system because of fragmentation and a lack of coordination, manual systems, complete or partial lack of automation and mixing of paper-based and computerised systems (South African National Department of Health 2019). In a similar setting it was suggested that a lack of availability of these clinical electronic systems, may have had an impact on perceived relevance and competence (Bhebe & De La Harpe 2014). This challenge may be addressed by providing nursing students with opportunities to practise and develop electronic documentation skills that they will use in practice using simulation and learning management systems (Bowling 2016). The respondents in this study have been exposed to both simulation experiences, as well as to teaching and learning opportunities via a learning management system, in their nursing programme; however, integrating clinical electronic documentation skills with classroom experiences should also be explored.

Informatics literacy and management skills are generally regarded as higher-level nursing informatics competence skills (Choi & De Martinis 2013) and nurses will not be able to effectively use health information technologies in nursing practice, if nursing informatics education is insufficient (Jouparinejad et al. 2020). Skills taught in classes can thus not be transferred well to the workplace, resulting in a lack of nursing informatics knowledge among graduating nurses if there are shortcomings in their education (Rajalahti et al. 2014).

### Attitudes towards computerisation

Overall, the respondents had positive attitudes towards computerisation, especially positive attitudes around reduced paperwork for nurses and ensuring efficiency in nursing care. This finding was similar to other studies were nurses were reported to have positive attitudes towards computers (Gurdas, Topkaya and Kaya, 2015) and where computer education and experience were significant factors contributing to these positive attitudes (Vijayalakshmi & Math, 2013). This finding is also consistent with a study in Jordan which showed that oncology nurses’ attitudes toward computerisation, particularly electronic health records were positive reflecting their awareness of the benefits of computers, and its relevance in nursing practice (Banihani and Al Qadire 2021). The negative attitudes towards computerisation in healthcare could be related to the low exposure to health informatics with poor exposure in clinical settings and a lack of formal informatics education in the respondents’ curriculum.

### Recommendations

#### Nursing education and practice

The introduction of formal nursing informatics training and the integration of nursing informatics skills as strands in clinical training will help prepare nursing students for the 4IR and the application and use in practice (Kaur & Rawat 2015). Especially, specific attention needs to be given to issues such as electronic health records and the use of new innovative devices in nursing practice. Equipping nursing students with the required knowledge and skills to use new and emerging digital tools will support their nursing practice within the clinical environment as a student, and result in competent nurse graduates entering the workplace (Collins et al. 2017).

#### Nursing research

With the advent of the coronavirus disease 2019 (COVID-19) pandemic and the 4IR, this study should be repeated with a bigger sample of both nursing students and practising nurses. The data should be anchored in a set of nursing informatics competencies relevant to this context and time and further studies on the perceived relevance and competence of practicing nurses on the nursing informatics will help to identify core nursing informatics competencies for professional nursing practice (Collins et al. 2017).

### Limitations

The study was limited to one school of nursing and thus cannot be generalised beyond the specific school. Some questions may have been difficult for respondents to complete and an adaptation of the questions to current practice may be beneficial.

## Conclusion

This study investigated final year nursing students’ perceptions on the relevance of nursing informatics skills along with their perceived competence in these skills and their attitudes towards computerisation. Although the educational preparation of the respondents in this study appears to successfully develop computer literacy skills, such as using a computer and accessing and using data, the development of nursing informatics literacy and management skills is inadequate.
